# Dermatologists’ Perceptions of the Use of Teledermatology in Managing Hidradenitis Suppurativa: Survey Study

**DOI:** 10.2196/43910

**Published:** 2023-01-31

**Authors:** Valencia Long, Ellie Ci-En Choi, Zhaojin Chen, Moonyza Akmal Ahmad Kamil, Murlidhar Rajagopalan, Erin McMeniman, Nisha Suyien Chandran

**Affiliations:** 1 Division of Dermatology, Department of Medicine, National University Hospital Singapore Singapore; 2 Biostatistics Unit Yong Loo Lin School of Medicine National University of Singapore Singapore Singapore; 3 Department of Dermatology Kuala Lumpur Hospital Kuala Lumpur Malaysia; 4 Department of Dermatology Apollo Hospitals Tamil Nadu India; 5 Department of Dermatology Princess Alexandra Hospital Brisbane Australia

**Keywords:** teledermatology, telehealth, telemedicine, hidradenitis suppurativa, atopic dermatitis, dermatitis, skin, dermatology, perception, experience, attitude, opinion, dermatologist, health care professional, health care provider, acceptance, adoption, COVID-19

## Abstract

**Background:**

The field of teledermatology has expanded tremendously and has been used for conditions including hidradenitis suppurativa (HS). However, due to the sensitive location of lesions, HS may be considered less suitable for teledermatology.

**Objective:**

We sought to assess dermatologists’ experiences and perceptions toward using teledermatology for HS relative to atopic dermatitis (AD) as a comparison.

**Methods:**

A survey was disseminated electronically to practicing dermatologists in the Asia-Pacific region between February and June 2022. Differences in attitudes and perceptions between HS and AD were compared using random-effects ordered logistic regression, controlling for demographics.

**Results:**

A total of 100 responses were obtained comprising of 76 (81.7%) dermatologists and 17 (18.3%) dermatology trainees; 62.6% (62/98) of physicians were uncomfortable with using teledermatology for HS. Multivariable regression confirmed increased perceived challenges with managing HS using teledermatology compared to AD. These challenges include the need for photography of hard-to-reach or sensitive areas (odds ratio [OR] 4.71, 95% CI 2.44-9.07; *P*<.001), difficulties in accurate assessment of severity (OR 2.66, 95%CI 1.48-4.79; *P*=.001), and inability to palpate lesions (OR 2.27, 95% CI 1.23-4.18; *P*=.009).

**Conclusions:**

This study confirms the relative reluctance of dermatologists to use teledermatology for HS and complements existing data showing mixed levels of willingness from patients. The use of teledermatology for HS may need to be optimized to overcome these challenges, including increasing security features, selection of patients with milder or limited diseases, and selecting patients with an established and strong doctor-patient relationship.

## Introduction

With the COVID-19 pandemic, there has been a rapid adoption of teledermatology on a global scale. A large survey study by the American Academy of Dermatology Teledermatology Task Force subgroup assessed the effects of COVID-19 on teledermatology among American Academy of Dermatology members, illuminating dermatologists’ sentiments toward different teledermatology modes as well as their opinions regarding reimbursement, perceived barriers, and anticipated future use [1].

Hidradenitis suppurativa (HS) is a condition that has been managed with teledermatology [2-4]; however, unique barriers and considerations may be present due to the sensitive sites and nature of skin lesions. Although several studies have explored the willingness and concerns of patients with HS toward teledermatology, the perceptions of dermatologists in this regard remains relatively unexplored.

## Methods

### Procedures

An electronic questionnaire was disseminated to members of the Asia Pacific Hidradenitis Suppurativa Foundation through a central email blast and word of mouth. Responses were gathered from February to June 2022. Inclusion criteria included any current practicing dermatologist (in private and public sectors) or dermatology trainee. Exclusion criteria included dermatologists who were not actively practicing and health care practitioners who were not yet officiated under a dermatology training program.

The survey questioned opinions toward using teledermatology to manage HS. To differentiate concerns specific to HS and those relating to teledermatology in general, perceptions were compared with atopic dermatitis (AD), a common chronic inflammatory dermatosis that has been managed over teledermatology. Several questions from this survey drew reference from the Likert-scale questions in the study by Kennedy et al [1]. Physician demographics, such as age, gender, and practice type, were also collected. Responses were anonymous and collected using a secure platform (FormSG).

Descriptive findings were summarized by frequency (percentages). Attitudes of physicians toward using teledermatology for HS and AD were summarized according to the extent of agreement (ie, strongly disagree, disagree, neither agree nor disagree, agree, and strongly agree). For simplicity of data representation, the groups were reduced to “agree,” “neutral,” and “disagree.” Associations between demographics and attitudes toward teledermatology for HS were evaluated by multivariable ordered logistic regression. The proportional odds assumption was assessed by approximate likelihood-ratio test via a generalized ordered logistic regression. Attitudes toward teledermatology between HS and AD were compared by random-effects ordered logistic regression, with adjustment for demographics. Age was treated as a continuous variable and analyzed at 5-year unit intervals. All statistical analyses were conducted using Stata/SE (version 17.0; StataCorp LLC). All statistical tests were 2-sided with a 5% significance level.

### Ethics Approval

This study was approved by the National Health Group institutional review board (DSRB 2021/00632). Respondents were not compensated for participating in this study.

## Results

In total, 100 responses were obtained, comprising of 76 (81.7%) dermatologists and 17 (18.3%) dermatology trainees. There was an equal representation of male and female genders (n=52, 52% males). The majority of physicians (n=70, 70%) were between 30-45 years of age, and 88 (86.3%) physicians practiced in parts of the Asia-Pacific region. More than half of the surveyed physicians (n=64, 64%) worked in public institutions (Table 1).

A minority of respondents (38/98, 38.8%) agreed or strongly agreed that there were comfortable using teledermatology to manage HS. The majority (62/98, 62.6%) disagreed or strongly disagreed that they would be comfortable using teledermatology to replace their usual physical HS consultations; only 30/98 (30.6%) perceived their patients with HS to be receptive toward teledermatology (Table 2).

Physician age influenced perceived comfort with using teledermatology for HS. Older physicians tended to express difficulties with accurate assessment of disease severity for HS over teledermatology (for every 5 year increment of age, there was an increased OR of 1.30, 95%CI 1.01-1.67; *P*=.045) and concerns that patients may not be familiar with using teledermatology for HS (for every 5 year increment of age, there was an increased OR of 1.37, 95%CI 1.05-1.80; *P*=.02; Table S1 in Multimedia Appendix 1).

**Figure 1:**
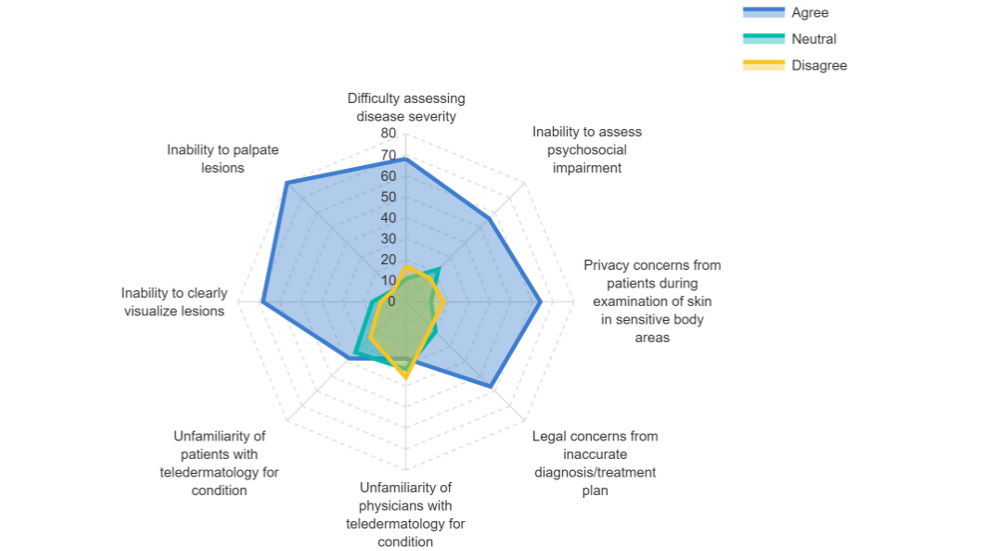
Challenges faced by physicians when using teledermatology for hidradenitis suppurativa.

**Figure 2:**
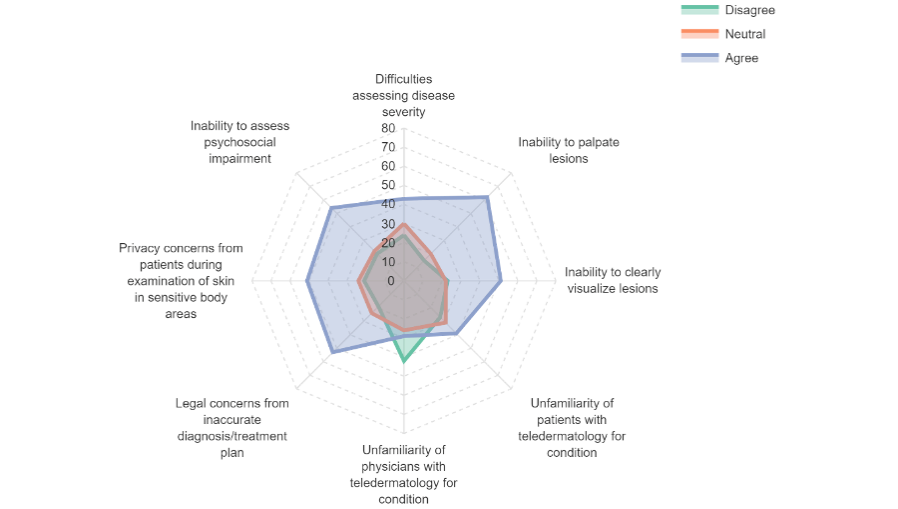
Challenges faced by physicians when using for atopic dermatitis.

Figure 1 and Figure 2 show the distribution of responses regarding the perceptions toward use of teledermatology for HS and AD, respectively. For both HS and AD, the greatest reported barriers toward the use of teledermatology were difficulties with assessing disease severity and inability to palpate lesions. In contrast, unfamiliarity of physicians followed by unfamiliarity of patients with teledermatology were the least reported barriers.

Multivariable regression confirmed increased perceived challenges with managing HS using teledermatology compared to AD even after controlling for physician demographics. These challenges include the following: (1) ensuring privacy when examining sensitive body areas (OR 2.75, 95%CI 1.36-5.56; *P*=.005); (2) photography of hard-to-reach or sensitive areas (OR 4.71, 95% CI 2.44-9.07; *P*<.001); (3) accurate assessment of severity (OR 2.66, 95%CI 1.48-4.79; *P*=.001); (4) ability to palpate lesions (OR 2.27, 95% CI 1.23-4.18; *P*=.009); and (5) visualization of lesions clearly over teledermatology (OR 3.59, 95% CI 1.86-6.96; *P*<.001; Table 3).

**Table 1:** Demographics of survey respondents.

Characteristics		Values, n (%)
**Age (years)**		
	30-35	26 (26)
	36-40	24 (24)
	41-45	20 (20)
	46-50	10 (10)
	51-55	2 (2)
	56-60	8 (8)
	61-65	4 (4)
	66-70	4 (4)
	>70	2 (2)
**Gender**		
	Male	52 (52)
	Female	48 (48)
**Type of practice**		
	Public or restructured	64 (64)
	Private hospital	10 (10)
	Private clinic (solo)	13 (13)
	Private clinic (group)	13 (13)
**Current role**		
	Dermatologist	76 (81.7)
	Dermatology trainee	17 (18.3)

**Table 2:** Physician views toward using teledermatology to manage hidradenitis suppurativa (HS).

Questionnaire items	Strongly disagree, n (%)	Disagree, n (%)	Neither agree nor disagree, n (%)	Agree, n (%)	Strongly agree, n (%)
I would feel comfortable using teledermatology to manage HS.	6 (6.1)	26 (26.5)	28 (28.6)	28 (28.6)	10 (10.2)
I would feel comfortable with using teledermatology to replace my usual consults for HS.	22 (22.2)	40 (40.4)	25 (25.3)	9 (9.1)	3 (3)
I think my patients with HS are generally technologically savvy.	4 (4.1)	22 (22.5)	51 (52)	14 (14.3)	7 (7.1)
I think my patients with HS would be receptive to being seen over teledermatology.	6 (6.1)	28 (28.6)	34 (34.7)	23 (23.5)	7 (7.1)
The keenness of my patient with HS to do a teledermatology would increase my willingness to do a teledermatology consult.	6 (6.1)	12 (12.2)	29 (29.6)	37 (37.8)	14 (14.3)

**Table 3:** Comparison of attitudes toward teledermatology between hidradenitis suppurativa (HS) and atopic dermatitis (AD). Italicized *P* values are significant.

HS vs AD	Univariable			Multivariable^a^	
	Unadjusted OR^b^ (95% CI)	*P* value	Adjusted OR (95% CI)		*P* value
It is difficult for patients to photograph or video hard-to-reach or sensitive areas	4.25 (2.28-7.94)	<.001	4.71 (2.44-9.07)		*<.001*
It is difficult for physician to accurately assess disease severity	2.72 (1.54-4.81)	.001	2.66 (1.48-4.79)		*.001*
Unable to palpate lesions over teledermatology	2.37 (1.30-4.32)	.005	2.27 (1.23-4.18)		*.009*
Unable to visualize lesions clearly over teledermatology	3.30 (1.75-6.20)	<.001	3.59 (1.86-6.96)		*<.001*
Concerns that patients may not be familiar with using teledermatology for condition	1.36 (0.73-2.54)	0.34	1.61 (0.83-3.12)		0.16
Concerns that physicians may not be familiar with using teledermatology for condition	1.24 (0.69-2.22)	0.47	1.22 (0.67-2.21)		0.51
Concerns about litigation regarding inaccurate diagnosis or treatment plan	1.39 (0.74-2.59)	0.30	1.41 (0.74-2.68)		0.30
Concerns about privacy issues arising in patients when examining skin in sensitive body areas	3.03 (1.51-6.06)	.002	2.75 (1.36-5.56)		*.005*
Inability to properly assess psychosocial state and impairment experienced by patients	1.68 (0.87-3.24)	0.12	1.71 (0.87-3.37)		0.20

^a^Age group, gender (female vs male), practice (private vs public), and role (trainee vs dermatologist) were adjusted in the multivariable analyses.

^b^OR: odds ratio.

## Discussion

### Principal Findings

Compared to other chronic, debilitating dermatological conditions—such as acne and AD, which have been managed via telemedicine during the COVID-19 pandemic—the role of teledermatology services for patients with HS has not been well characterized.

HS is unique compared to other chronic dermatological conditions due to its propensity for skin lesions that involve deeper layers of skin and of largely intimate body areas. This highlights the need for an independent investigation into the barriers to teledermatology for HS.

Our study findings confirm that the use of teledermatology for patients with HS lacks strong traction among dermatologists. We further demonstrate that a significant proportion of physicians were reluctant to have teledermatology completely replace their routine face-to-face visits and were ambivalent toward perceived willingness of patients to be managed over teledermatology. This reluctance was more apparent in older physicians who perceived more difficulties with accurate assessment of disease severity and had concerns that patients with HS may not be familiar with the technology. This echoes the findings of Choi et al’s [5] mixed methods study on the use and perceptions of teledermatology in 942 Asian patients, which showed that age (or youth) was independently associated with greater willingness to use teledermatology. Our study shows that age is a common factor influencing individuals’ (be it physician or patient) willingness to engage in teledermatology.

Furthermore, the apparent reluctance portrayed by physicians in this study could be associated with time period bias. During the peak of the COVID-19 pandemic, when there were strict lockdowns, teledermatology was positioned as one of the few available means to obtain a health consult. In comparison, a year after the onset of the COVID-19 pandemic, when this survey was conducted, many health care systems have seen a gradual lifting of physical isolation policies, allowing physical consultations to take place. The overall willingness of health care providers to practice teledermatology revealed in those studies may therefore have been overinflated [5-7].

We found significant differences in attitudes and perspectives for physicians in terms of using teledermatology to manage HS compared to AD, with overall increased tendency for physicians to experience difficulty in managing HS compared to AD. Most concerns revolved around perceived difficulty for patients with HS in photographing or videoing hard-to-reach or sensitive areas and physician-reported difficulties with accurate assessment of disease severity for HS compared to AD—consistent with existing literature expounding the challenges faced by dermatologists when providing teledermatology for HS [3,8]. With AD being one of the commonest chronic skin conditions, priority for the optimization of teledermatology for its diagnosis and management has enabled more widespread use with provisions for in-person appointments with dermatologists for most of the severe cases [9,10]. In recent years, studies have also given support to the use of telemedicine in treating patients with AD [11-14]. In comparison, the complex nature of HS management has impeded more rapid use of teledermatology. Andriano et al [15] highlighted that those patients with HS who were satisfied with teledermatology tended to have mild disease and were less likely to require office visits for acute flare management. It is likely that physicians would think alike and may be more cautious in their overall outlook of the usefulness of teledermatology for patients with a more severe HS.

Finally, this study raises significant clinical considerations for the use of teledermatology in patients with HS, particularly as the COVID-19 pandemic evolves and the need for strict physical isolation and social distancing is reduced. Ambivalence of physicians regarding uptake suggests a need to continually optimize teledermatology to ensure sustainability. We suggest that physicians could more strongly consider offering such services to patients with quiescent or mild HS (ie, Hurley stage I, International Hidradenitis Suppurativa Severity Score System score 1-3); patients with less HS involvement in intimate body areas; patients who are more willing to share documentation, if required, of affected body areas; and patients who already have an established and strong doctor-patient relationship with their HS provider. The use of teledermatology for HS is also helpful in circumstances of surges in outpatient and inpatient attendances, where physical clinical space is limited. The use of specialized, secure telehealth platforms may boost physicians’ confidence to manage HS and patient’s assurance of data security; this includes the telehealth module from the Epic Systems Corporation (or EPIC)—a largely nationwide, secure clinical records platform used in Singapore. Cultural barriers require navigation, as examination of sensitive body parts (such as genitalia) over teledermatology might still be challenging in more conservative regions [16-18]. This echoes the findings by Okeke et al [8], who suggest that the act of requesting for patient representation via technology—be it for patient-submitted photographs or real-time video examination of HS-affected skin in teleconsultations, where physician-patient rapport is harder to establish—could generate significant patient unease. Difficulties with navigating cultural barriers, coupled with the restoration of face-to-face consults as the region emerges from the pandemic, may impede robust teledermatology uptake for HS in the Asia-Pacific region.

Learning from previously published literature and cautionary messages for practicing physicians could streamline the teledermatology practice for patients with HS. In his study of 41 patients with HS who were being treated over teledermatology, Patel [19] has described that the center’s approach was to request images of HS-affected skin only when deemed “essential,” such as prior to urgent commencement of adalimumab. Patel further stressed the importance of clinical face-to-face assessment, advising the need to handle photographic or video evaluation sensitively due to the high prevalence of anxiety and depression in the affected patient. We suggest that it would be reasonable to first assess the likely severity of HS in patients who are managed over teledermatology, through sensitive history taking—triaging patients who may require urgent commencement of biologics or conversion to face-to-face consultations to assess flare symptoms and those who have previously tried other forms of treatment and have failed to respond. Assessing patients’ level of psychoemotional concerns (such as anxiety, depression, and body dysmorphism) may also help physicians select suitable modes of teledermatology consults, be it hybrid, video- or telephone-based, or other modes.

Although many studies have exhorted the benefits of teledermatology, we recommend that physicians need to remain vigilant about the nuances of this practice and continue to refine the service for HS with the above-suggested patient profiles and caveats.

Strengths of this study include a fairly large sample size, including global respondents, with a predominance of respondents from the Asia-Pacific region. The inclusion of a compactor condition (ie, AD) provided a reference with which perceptions toward teledermatology use in HS could be compared.

Limitations of this study include inability to assess true response rates (as the survey was disseminated to respondents who had the option of further eligible colleagues) and potential response bias. Teledermatology as a term is also broad and encompasses various modalities (eg, videos; telephone calls; as well as store and forward modalities, which use electronically stored health information, including patient photographs, for clinical decision-making asynchronous to the patient encounter). Further questionnaires could investigate differential perceptions and comfort with regard to various modalities. There is also limited generalizability of findings to the Asia-Pacific region.

### Conclusions

Our study suggests that dermatologists in Asia find HS difficult to manage via teledermatology, especially in comparison to AD. However, teledermatology in this region may be considered useful in selected settings. Physicians’ efforts should be focused on streamlining patient selection and optimizing consult environments for patients with HS.

## Appendix

Multimedia Appendix 1 Association between demographics and attitudes of physicians towards teledermatology for hidradenitis suppurativa.

## Abbreviations

AD: atopic dermatitis
HS: hidradenitis suppurativa
OR: odds ratio
