# Supplementary material for: Dermatologists’ Perceptions of the Use of Teledermatology in Managing Hidradenitis Suppurativa: Survey Study
Source: JMIR Dermatol. 2023 Jan 31;6:e43910. doi: 10.2196/43910 (PMC10335133; doi:10.2196/43910)
Supplement: Multimedia Appendix 1 [file derma_v6i1e43910_app1.docx]

**Table S1:** Association between demographics and attitudes of physicians towards teledermatology for hidradenitis suppurativa

|  | **Adjusted OR* (95% CI)** | ***p*-value** |
| --- | --- | --- |
| *I would feel comfortable using teledermatology to manage HS* | | |
| Age group | 1.15 (0.90-1.49) | 0.266 |
| Gender (female vs. male) | 0.95 (0.41-2.16) | 0.894 |
| Practice (private vs. public) | 1.41 (0.51-3.85) | 0.506 |
| Role (trainee vs. dermatologist) | 1.08 (0.39-3.02) | 0.881 |
| *I would feel comfortable with using teledermatology to replace my usual consults for HS* | | |
| Age group | 0.87 (0.68-1.10) | 0.238 |
| Gender (female vs. male) | 1.17 (0.53-2.62) | 0.695 |
| Practice (private vs. public) | 0.83 (0.32-2.17) | 0.706 |
| Role (trainee vs. dermatologist) | 1.13 (0.39-3.31) | 0.824 |
| *I think my HS patients are generally technologically savvy* | | |
| Age group | 0.89 (0.70-1.14) | 0.363 |
| Gender (female vs. male) | 1.50 (0.65-3.49) | 0.343 |
| Practice (private vs. public) | 1.23 (0.45-3.37) | 0.682 |
| Role (trainee vs. dermatologist) | 0.66 (0.22-1.97) | 0.459 |
| *I think my HS patients would be receptive to being seen over teledermatology* | | |
| Age group | 0.87 (0.68-1.12) | 0.292 |
| Gender (female vs. male) | 0.80 (0.36-1.79) | 0.594 |
| Practice (private vs. public) | 0.83 (0.32-2.13) | 0.695 |
| Role (trainee vs. dermatologist) | 0.56 (0.20-1.58) | 0.274 |
| *The keenness of my HS patient to do a teledermatology consult would increase my willingness to do a teledermatology consult* | | |
| Age group | 1.16 (0.91-1.48) | 0.234 |
| Gender (female vs. male) | 1.47 (0.66-3.27) | 0.351 |
| Practice (private vs. public) | 1.48 (0.55-4.00) | 0.438 |
| Role (trainee vs. dermatologist) | 1.55 (0.55-4.39) | 0.405 |
| *It is difficult for patients to photograph or video hard-to-reach/sensitive areas* | | |
| Age group | 1.09 (0.85-1.40) | 0.500 |
| Gender (female vs. male) | 0.76 (0.34-1.74) | 0.522 |
| Practice (private vs. public) | 0.64 (0.24-1.69) | 0.367 |
| Role (trainee vs. dermatologist) | 1.00 (0.34-2.97) | 0.996 |
| *It is difficult for physician to accurately assess disease severity* | | |
| Age group | 1.30 (1.01-1.67) | **0.045^†^** |
| Gender (female vs. male) | 0.77 (0.34-1.78) | 0.549 |
| Practice (private vs. public) | 0.51 (0.19-1.33) | 0.168 |
| Role (trainee vs. dermatologist) | 1.39 (0.44-4.34) | 0.573 |
| *Unable to palpate lesions over teledermatology* |  |  |
| Age group | 1.30 (0.99-1.70) | 0.062 |
| Gender (female vs. male) | 0.70 (0.30-1.64) | 0.416 |
| Practice (private vs. public) | 0.49 (0.18-1.33) | 0.163 |
| Role (trainee vs. dermatologist) | 0.52 (0.17-1.59) | 0.252 |
| *Unable to visualize lesions clearly over teledermatology* | | |
| Age group | 1.27 (0.98-1.64) | 0.068 |
| Gender (female vs. male) | 0.51 (0.22-1.20) | 0.122 |
| Practice (private vs. public) | 0.36 (0.14-0.95) | **0.039^†^** |
| Role (trainee vs. dermatologist) | 0.78 (0.26-2.35) | 0.661 |
| *Concerns that patients may not be familiar with using teledermatology for condition* | | |
| Age group | 1.37 (1.05-1.80) | **0.021^†^** |
| Gender (female vs. male) | 0.65 (0.29-1.48) | 0.304 |
| Practice (private vs. public) | 0.62 (0.23-1.66) | 0.342 |
| Role (trainee vs. dermatologist) | 2.37 (0.81-6.96) | 0.117 |
| *Concerns that physicians may not be familiar with using teledermatology for condition* | | |
| Age group | 1.11 (0.88-1.40) | 0.390 |
| Gender (female vs. male) | 0.74 (0.33-1.66) | 0.468 |
| Practice (private vs. public) | 1.11 (0.43-2.86) | 0.837 |
| Role (trainee vs. dermatologist) | 2.09 (0.73-5.99) | 0.168 |
| *Concerns about litigation regarding inaccurate diagnosis/treatment plan* | | |
| Age group | 1.14 (0.89-1.47) | 0.298 |
| Gender (female vs. male) | 0.66 (0.29-1.53) | 0.331 |
| Practice (private vs. public) | 1.08 (0.41-2.82) | 0.879 |
| Role (trainee vs. dermatologist) | 1.89 (0.61-5.81) | 0.269 |
| *Concerns about privacy issues arising in patients when examining skin in sensitive body areas* | | |
| Age group | 1.17 (0.90-1.51) | 0.236 |
| Gender (female vs. male) | 0.91 (0.40-2.08) | 0.819 |
| Practice (private vs. public) | 0.85 (0.32-2.26) | 0.751 |
| Role (trainee vs. dermatologist) | 1.75 (0.59-5.23) | 0.316 |
| *Inability to properly assess psycho-social state and impairment experienced by patients* | | |
| Age group | 1.19 (0.93-1.52) | 0.174 |
| Gender (female vs. male) | 0.91 (0.39-2.09) | 0.820 |
| Practice (private vs. public) | 1.97 (0.72-5.39) | 0.189 |
| Role (trainee vs. dermatologist) | 1.60 (0.54-4.74) | 0.398 |

* Age group, gender (female vs. male), practice (private vs. public) and role (trainee vs. dermatologist) were adjusted. Age was treated as a continuous variable with a 5-year unit interval
**^†^** Significant values
